# Supplementary material for: The AAV capsid can influence the epigenetic marking of rAAV delivered episomal genomes in a species dependent manner
Source: Nat Commun. 2023 Apr 28;14:2448. doi: 10.1038/s41467-023-38106-3 (PMC10147666; doi:10.1038/s41467-023-38106-3)
Supplement: Supplementary file 1 — Supplementary Information [file 41467_2023_38106_MOESM1_ESM.pdf]

Supplementary Information

The AAV capsid can influence the epigenetic marking of rAAV delivered episomal genomes in a species dependent manner

Supplementary Figures 1-9

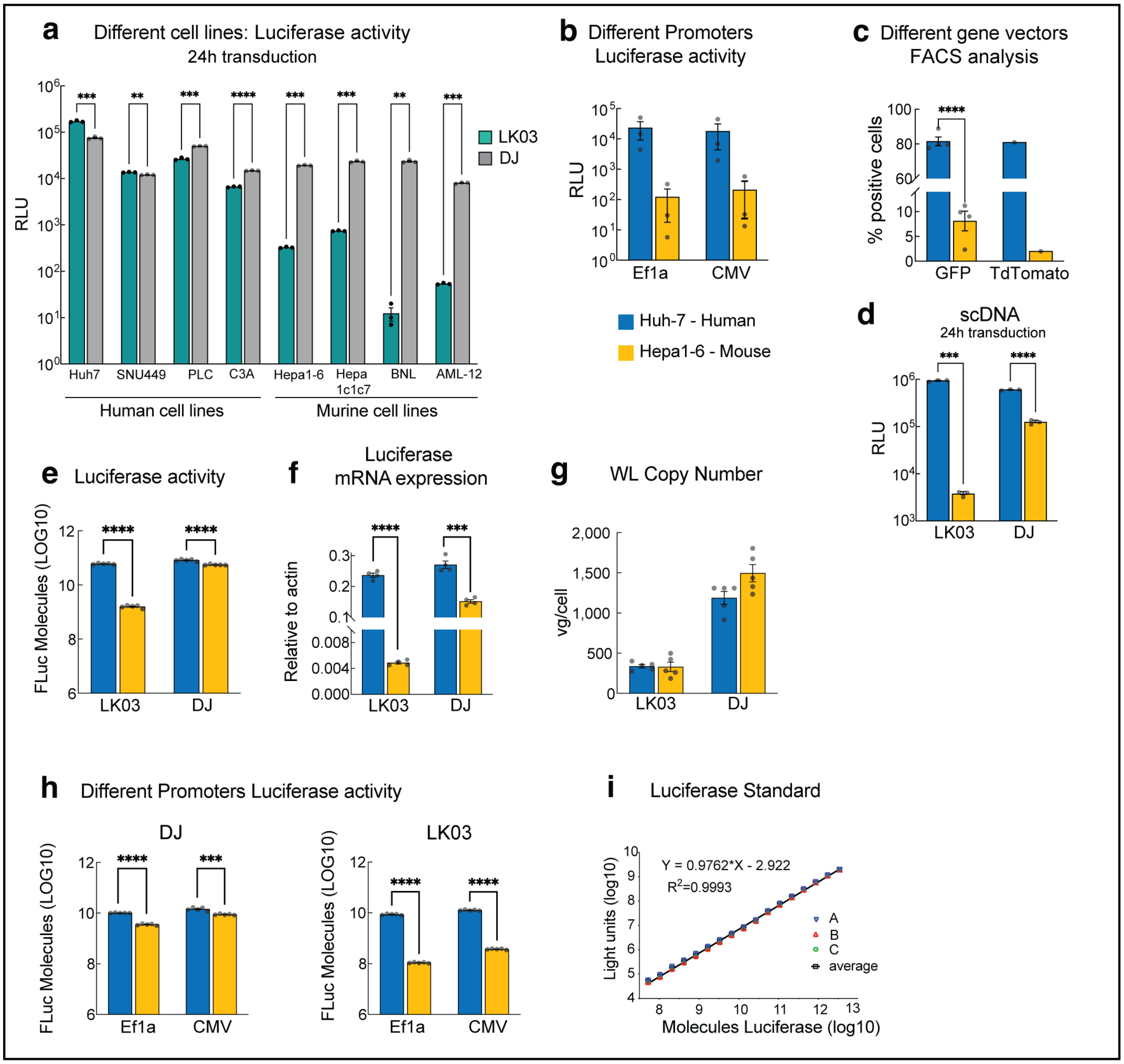

a Different cell lines: Luciferase activity
24h transduction
RLU
LK03
DJ
Huh7
SNU449
PLC
C3A
Hepa1-6
Hepa 1c1c7
BNL
AML-12
Human cell lines
Murine cell lines
b Different Promoters
Luciferase activity
RLU
Ef1a
CMV
Huh-7 - Human
Hepa1-6 - Mouse
c Different gene vectors
FACS analysis
% positive cells
GFP
TdTomato
d scDNA
24h transduction
RLU
LK03
DJ
e Luciferase activity
FLuc Molecules (LOG10)
LK03
DJ
f Luciferase
mRNA expression
Relative to actin
LK03
DJ
g WL Copy Number
vg/cell
LK03
DJ
h Different Promoters Luciferase activity
DJ
FLuc Molecules (LOG10)
Ef1a
CMV
LK03
FLuc Molecules (LOG10)
Ef1a
CMV
i Luciferase Standard
Y = 0.9762*X - 2.922
R2=0.9993
A
B
C
average
Light units (log10)
Molecules Luciferase (log10)

Supplementary Fig. 1: Transduction inefficiency of AAV-LK03 in murine cells is independent of cell line, promoter, transgene, or genome structure (scDNA). (**a**) Luciferase activity assay in various human and murine cell lines 24h post transduction using rAAV expressing FLuc cassette. (**b**) Luciferase activity in indicated cell lines 48h post transduction with AAV-LK03 packaged luciferase vectors with two different promoters (Ef1a or CMV). (**c**) FACS analysis of two cell lines 48h post transduction with AAV-LK03 expressing GFP or TdTomato under control of the CAG promoter. See Supplementary Fig. 9 for the gating strategy used. (**d**) Luciferase activity assay of cells 24h post transduction with indicated capsids delivering a self-complementary (sc) RLuc vector. (**e**) Luciferase activity in indicated cell lines 48h post transduction with AAV-DJ and AAV-LK03 packaged luciferase vectors with the CAG promoter. (**f**) Luciferase mRNA expression. (**g**) Whole lysate copy number. (**h**) Luciferase activity in indicated cell lines 48h post transduction with AAV-DJ and AAV-LK03 packaged luciferase vectors with two different promoters (Ef1a or CMV). (**i**) Standard curve for the luciferase assay performed for (**e**) and (**h**). Graphs e-h display results from an independent repeat experiment. Statistics were performed using multiple unpaired Welch t tests. For TdTomato in (**c**) statistsics were not performed since there was only one replicate. Only statistically significant differences are indicated. Statistic p-value * <0.05, ** <0.01, *** <0.001, **** <0.0001. . N >= 3 biologically independent samples, except for (**c**). Data are presented as mean values +/- SEM. Raw data for the graphs are provided in Source Data File 1. Detailed statistics for each graph are provided in Source Data File 2.

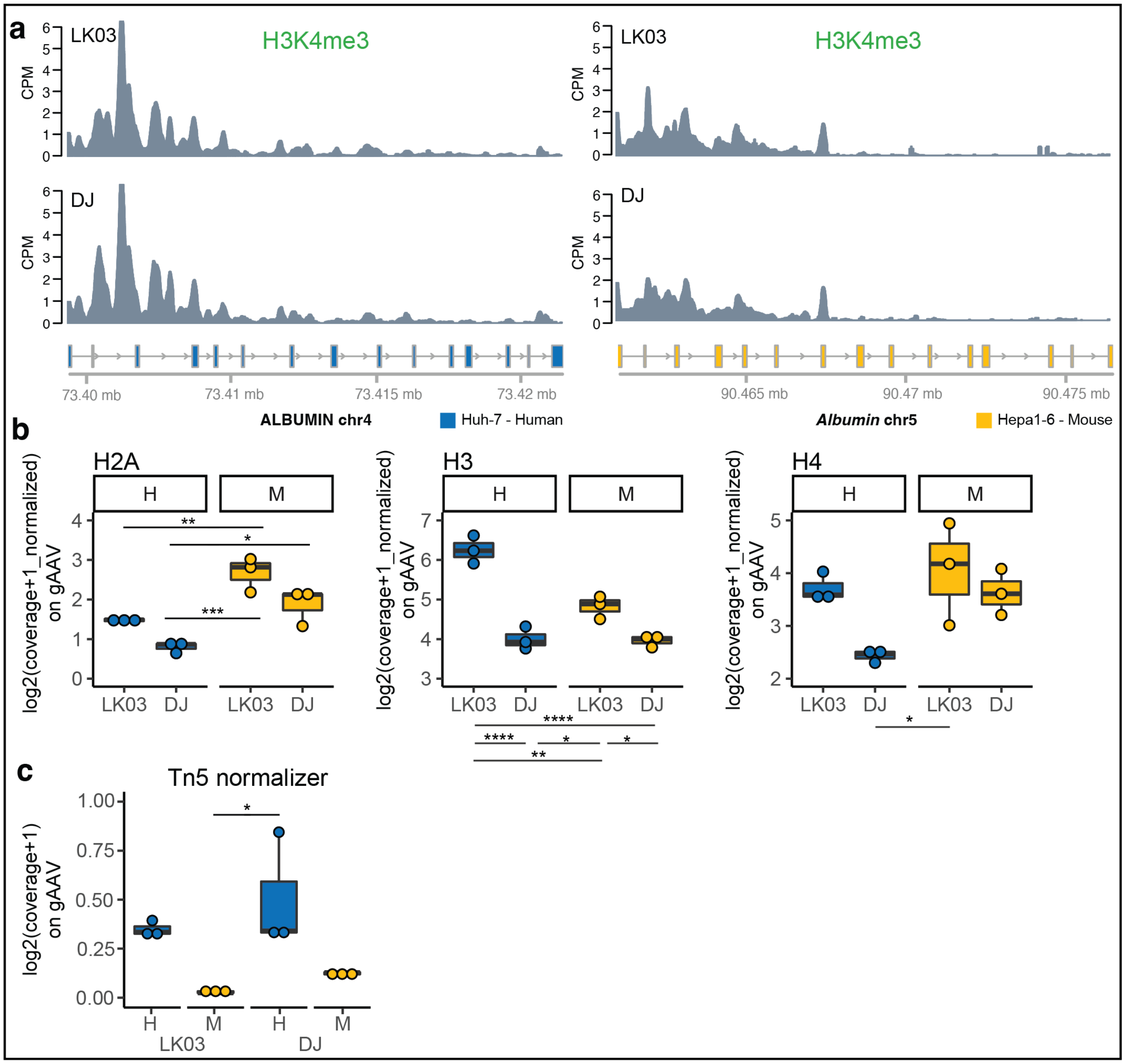
a
H3K4me3
LK03
DJ
CPM
73.40 mb
73.41 mb
73.415 mb
73.42 mb
90.465 mb
90.47 mb
90.475 mb
ALBUMIN chr4
Huh-7 - Human
Albumin chr5
Hepa1-6 - Mouse
b
H2A
H3
H4
H
M
log2(coverage+1_normalized) on gAAV
LK03
DJ
c
Tn5 normalizer
log2(coverage+1) on gAAV
1.00
0.75
0.50
0.25
0.00

Supplementary Fig. 2: Host genome H3K4me3 signals are comparable and independent of AAV capsid while core histones enrichment in gAAV is variable between capsids and species. (**a**) H3K4me3 normalized (CPM) signal on the *Albumin* gene (as representation of host genome) obtained from the Cut&Tag assay and next generation sequencing for Huh7 human and Hepa 1-6 mouse cell lines transduced with AAV-LK03 and AAV-DJ. (**b**) Cut&Tag boxplots of normalized coverage on the AAV genome, for core histones in Huh7 human and Hepa 1-6 mouse cell lines transduced with AAV-LK03 and AAV-DJ. (**c**) Tn5 Illumina DNA sequencing used as a normalizer value for Cut&Tag coverage for AAV-LK03 and AAV-DJ delivered gAAV. Statistics were performed using a 2 way ANOVA and only statistically significant differences are indicated. Statistic p-value * <0.05, ** <0.01, *** <0.001, **** <0.0001. H=human, M=mouse. . N >= 3 biologically independent samples. Boxplots display the median (thick bar), two hinges (lower and upper hinges correspond to the first and third quartiles) and two whiskers. The upper whisker extends from the hinge to the largest value no further than 1.5 *IQR (inter-quantile range) from the hinge. The lower whisker extends from the hinge to the smallest value at most 1.5 * IQR of the hinge. Raw data for the graphs are provided in Source Data File 1. Detailed statistics for each graph are provided in Source Data File 2.

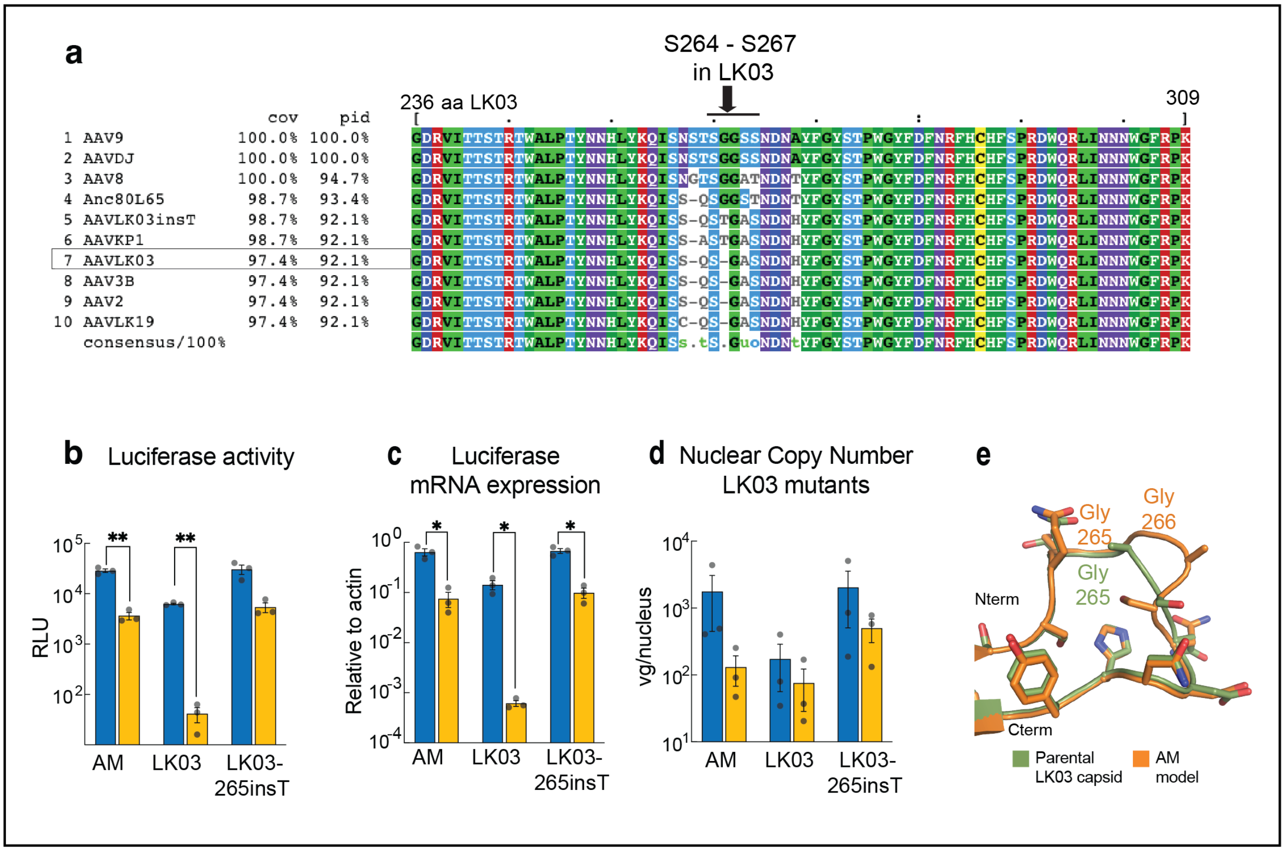
a
S264 - S267
in LK03
236 aa LK03
309
cov pid
1 AAV9 100.0% 100.0%
2 AAVDJ 100.0% 100.0%
3 AAV8 100.0% 94.7%
4 Anc80L65 98.7% 93.4%
5 AAVLK03insT 98.7% 92.1%
6 AAVKP1 98.7% 92.1%
7 AAVLK03 97.4% 92.1%
8 AAV3B 97.4% 92.1%
9 AAV2 97.4% 92.1%
10 AAVLK19 97.4% 92.1%
consensus/100%
b Luciferase activity
RLU
AM
LK03
LK03-265insT
c Luciferase mRNA expression
Relative to actin
d Nuclear Copy Number LK03 mutants
vg/nucleus
e
Gly 265
Gly 266
Gly 265
Nterm
Cterm
Parental LK03 capsid
AM model

Supplementary Fig. 3: AAV-AM is a capsid with an insertion of a glycine amino acid in the AAV-LK03 capsid sequence. Inactive-related histone modifications are similarly enriched, and core histones have variable enrichments, on genomes delivered by AAV-AM as the other capsids. (**a**) Alignment of AAV-LK03 with multiple other capsids from amino acid 236-309, arrow points at variable region where Glycine was inserted at position 265 to create capsid AAV-AM. Huh7 human and Hepa 1-6 mouse cells were transduced with AAV-AM, AAV-LK03 and AAV-LK03-265insT. (**b**) Luciferase activity quantification. (**c**) Relative quantification of Luciferase mRNA by qRT-PCR. (**d**) Nuclear copy number qPCR quantification. Data for AAV-AM and AAV-LK03 are identical to those shown in Fig. 3 a-c and are included here only for comparison reasons. (**e**) Homology model built using SwissModel server using the 3kie crystal structure as template (Parental AAV-LK03 capsid - AAV3B). Statistics were performed using multiple unpaired Welch t tests. Statistic p-value * <0.05, ** <0.01. . N >= 3 biologically independent samples. Data are presented as mean values +/- SEM. Raw data for the graphs are provided in Source Data File 1. Detailed statistics for each graph are provided in Source Data File 2.

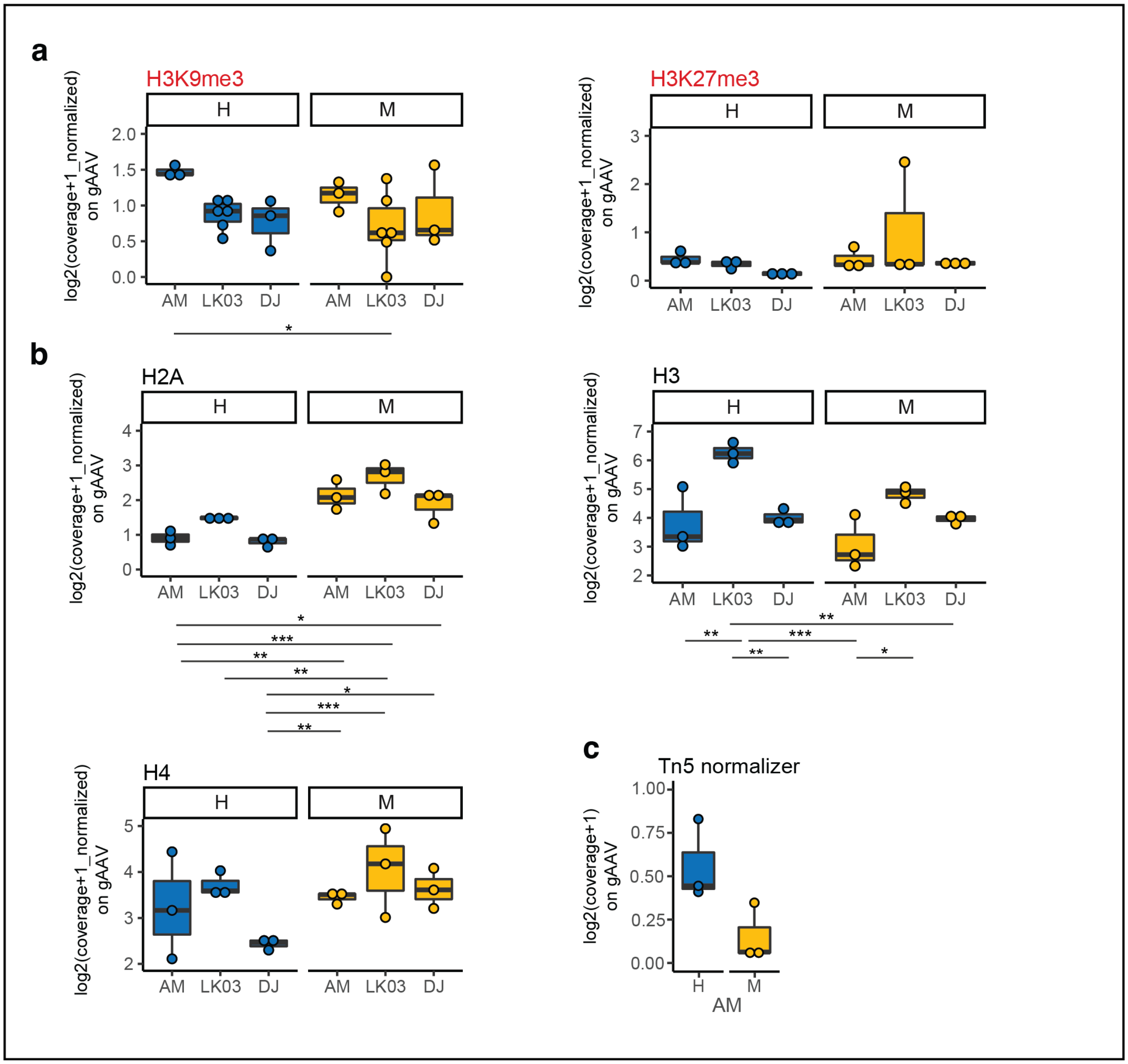

a
H3K9me3
H
M
log2(coverage+1_normalized) on gAAV
2.0
1.5
1.0
0.5
0.0
AM LK03 DJ AM LK03 DJ
*
H3K27me3
3
2
1
0
b
H2A
4
3
2
1
0
*
***
**
**
*
***
**
H3
7
6
5
4
3
2
**
**
***
**
*
H4
5
4
3
2
c
Tn5 normalizer
log2(coverage+1) on gAAV
1.00
0.75
0.50
0.25
0.00
H M
AM

Supplementary Fig. 4: Inactive-related histone modifications are similarly enriched, and core histones have variable enrichments, on genomes delivered by AAV-AM as the other capsids. (**a**) Cut&Tag boxplots of normalized coverage on AAV genome, for inactive-related histone modifications (H3K9me3, H3K27me3) and (**b**) core histones (H2A, H3, H4) for Huh7 human and Hepa 1-6 mouse cells transduced with AAV-AM, AAV-LK03 and AAV-DJ for 48h. (**c**) Tn5 Illumina DNA sequencing, to use as normalizer value for Cut&Tag coverage for AAV-AM delivered gAAV. Statistics were performed using a 2 way ANOVA except for (c) where an unpaired t test was used for analysis. Only statistically significant differences are indicated. Statistic p-value * <0.05, ** <0.01, *** <0.001. H=human, M=mouse. . N >= 3 biologically independent samples. Boxplots display the median (thick bar), two hinges (lower and upper hinges correspond to the first and third quartiles) and two whiskers. The upper whisker extends from the hinge to the largest value no further than 1.5 *IQR (inter-quantile range) from the hinge. The lower whisker extends from the hinge to the smallest value at most 1.5 * IQR of the hinge. Raw data for the graphs are provided in Source Data File 1. Detailed statistics for each graph are provided in Source Data File

2.

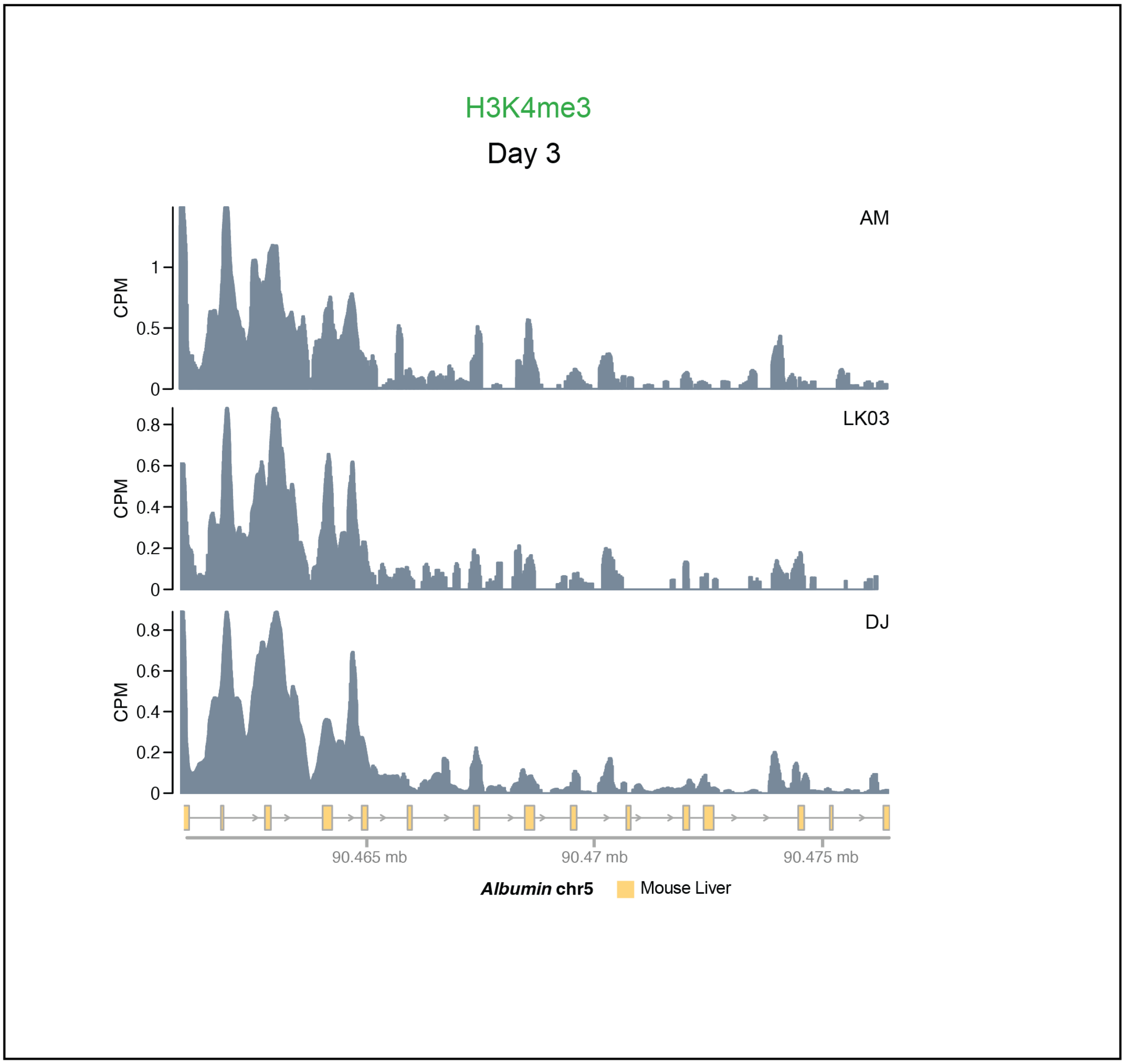

H3K4me3
Day 3
AM
LK03
DJ
CPM
0
0.5
1
0.2
0.4
0.6
0.8
90.465 mb
90.47 mb
90.475 mb
Albumin chr5
Mouse Liver

Supplementary Fig. 5: Host genome H3K4me3 signals are comparable independent of AAV transduction in vivo. Inactive-related histone marks and core histones enrichments are comparable between AAV-AM and AAV-DJ but not AAV-LK03 delivered genomes, in vivo. (**a**) H3K4me3 normalized (CPM) signal on the Albumin gene (as representation of the host genome) obtained from Cut&Tag assay and next generation sequencing for mouse liver transduced with AAV-AM, AAV-LK03 and AAV-DJ, collected 3 days post injection.

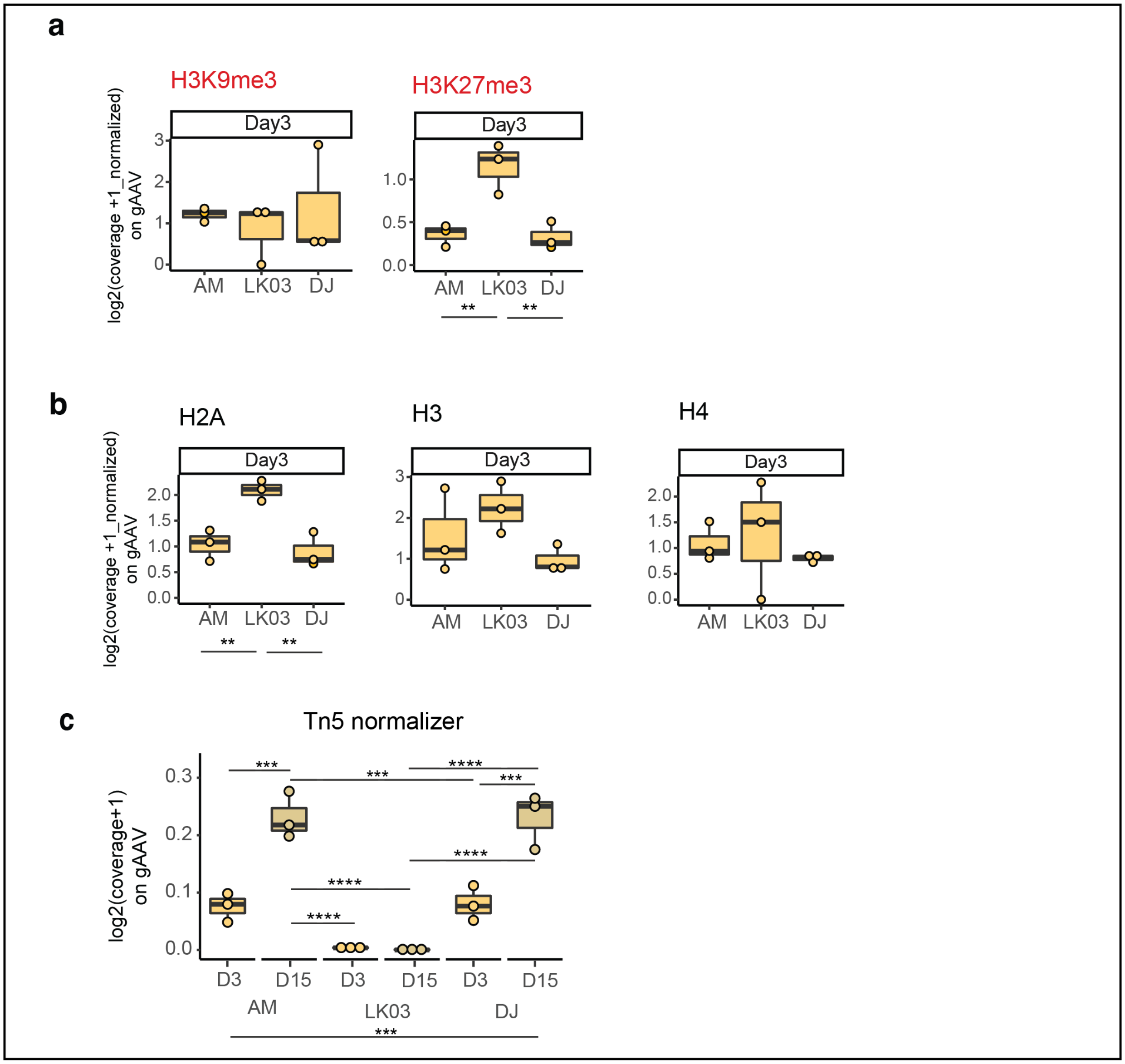

a
H3K9me3
H3K27me3
Day3
log2(coverage +1_normalized) on gAAV
AM LK03 DJ
**
b
H2A
H3
H4
c
Tn5 normalizer
log2(coverage+1) on gAAV
D3 D15 D3 D15 D3 D15
AM LK03 DJ
***
****

Supplementary Fig. 6: Host genome H3K4me3 signals are comparable independent of AAV transduction in vivo. Inactive-related histone marks and core histones enrichments are comparable between AAV-AM and AAV-DJ but not AAV-LK03 delivered genomes in vivo. (**a**) Cut&Tag boxplots of normalized coverage on AAV genome, for inactive-related histone modifications (H3K9me3, H3K27me3) and (**b**) core histones (H2A, H3, H4) for mouse liver transduced with AAV-AM, AAV-LK03 and AAV-DJ, collected 3 days post injection. (**c**) Tn5 Illumina DNA sequencing used as a normalizer value for Cut&Tag coverage for AAV genomes. For (a) and (b) statistics were performed using an ordinary one way ANOVA, for (C) 2 way ANOVA was used. Only statistically significant differences are indicated. Statistic p-value ** <0.01, *** <0.001, **** <0.0001. . N >= 3 biologically independent samples. Boxplots display the median (thick bar), two hinges (lower and upper hinges correspond to the first and third quartiles) and two whiskers. The upper whisker extends from the hinge to the largest value no further than 1.5 *IQR (inter-quantile range) from the hinge. The lower whisker extends from the hinge to the smallest value at most 1.5 * IQR of the hinge. Raw data for the graphs are provided in Source Data File 1. Detailed statistics for each graph are provided in Source Data File 2.

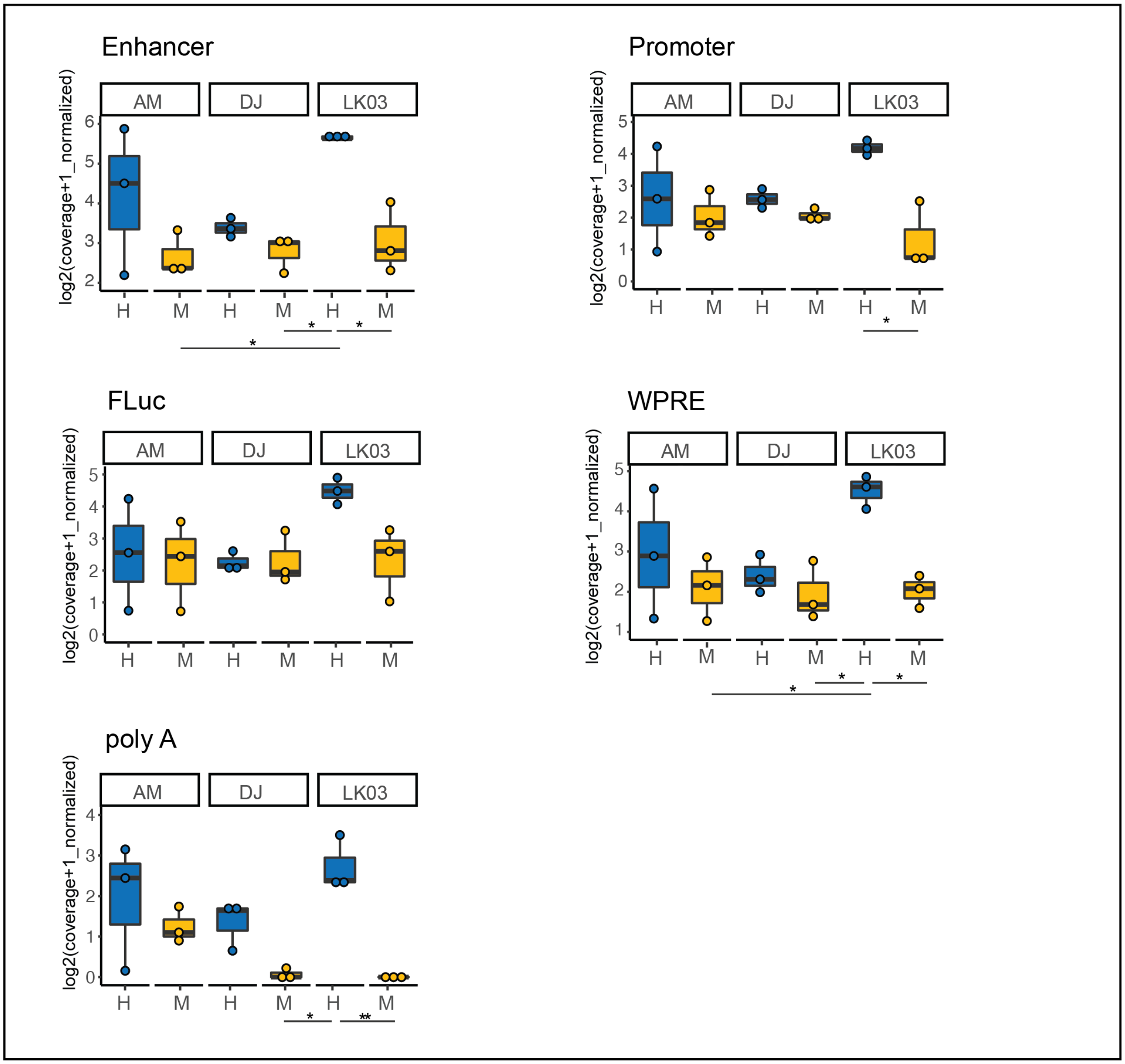

Enhancer
Promoter
FLuc
WPRE
poly A
AM
DJ
LK03
log2(coverage+1_normalized)
H
M
*
**

Supplementary Fig. 7: Higher rate of occupancy of Pol II across the entire AAV genome when delivered with LK03 capsid. Cut&Tag boxplots of normalized coverage on indicated AAV genome regions for Pol II in Huh7 human and Hepa 1-6 mouse cells transduced with AAV-AM, AAV-LK03 and AAV-DJ for 48h. Statistics were performed using a 2 way ANOVA. Only statistically significant differences are indicated. Statistic p-value * <0.05, ** <0.01. H=human, M=mouse. . N >= 3 biologically independent samples. Boxplots display the median (thick bar), two hinges (lower and upper hinges correspond to the first and third quartiles) and two whiskers. The upper whisker extends from the hinge to the largest value no further than 1.5 *IQR (inter-quantile range) from the hinge. The lower whisker extends from the hinge to the smallest value at most 1.5 * IQR of the hinge. Raw data for the graphs are provided in Source Data File 1. Detailed statistics for each graph are provided in Source Data File 2.

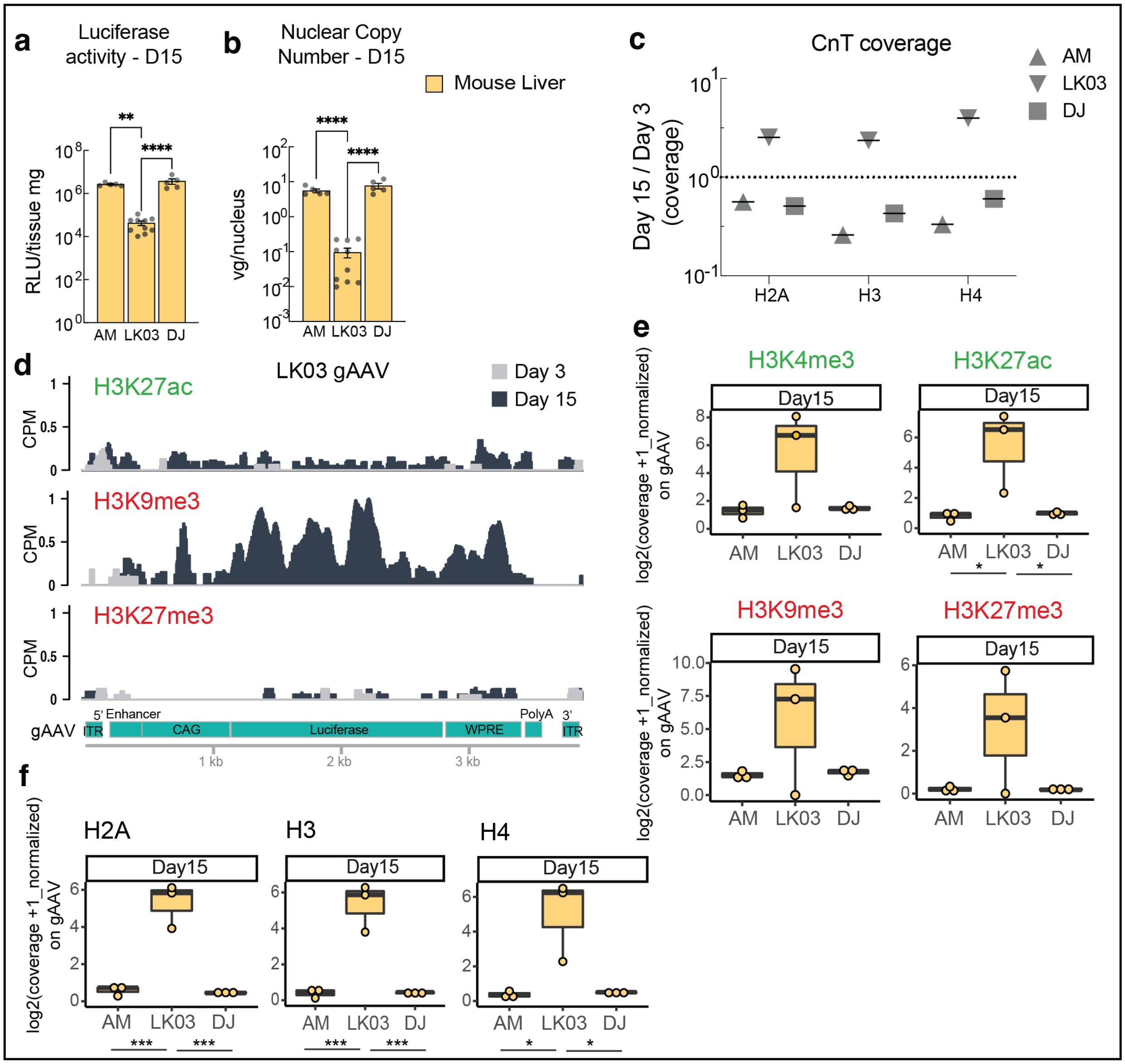

a
Luciferase activity - D15
RLU/tissue mg
AM LK03 DJ
b
Nuclear Copy Number - D15
vg/nucleus
AM LK03 DJ
Mouse Liver
c
CnT coverage
Day 15 / Day 3 (coverage)
H2A H3 H4
AM
LK03
DJ
d
LK03 gAAV
Day 3
Day 15
H3K27ac
H3K9me3
H3K27me3
CPM
gAAV
5' ITR
Enhancer
CAG
Luciferase
WPRE
PolyA
3' ITR
1 kb
2 kb
3 kb
e
H3K4me3
H3K27ac
H3K9me3
H3K27me3
Day15
log2(coverage +1_normalized) on gAAV
AM LK03 DJ
f
H2A
H3
H4
Day15
log2(coverage +1_normalized) on gAAV
AM LK03 DJ

Supplementary Fig. 8: *In vivo* assays on mouse liver tissue at 15 days post injection. (**a**) Luciferase activity, (**b**) nuclear copy number qPCR of AAV genomes as indicated, collected 15 days post injection. (**c**) Ratio Day 15 vs Day 3 post injection of Tn5 normalized Cut&Tag coverage for core histones. (**d**) Comparison of Cut&Tag enrichment of indicated histone modifications on AAV-LK03 derived AAV genome at different days post injection. Normalization as CPM. (**e**) Cut&Tag boxplots of normalized coverage on AAV genome of histone marks and (**f**) core histones, collected 15 days post injection. Statistics were performed using an ordinary one way ANOVA and only statistically significant differences are indicated for e and f. Statistic p-value * <0.05, ** <0.01, *** <0.001, **** <0.0001. N >= 3 biologically independent samples. Data are presented as mean values +/- SEM in bargraphs. Boxplots display the median (thick bar), two hinges (lower and upper hinges correspond to the first and third quartiles) and two whiskers. The upper whisker extends from the hinge to the largest value no further than 1.5 *IQR (inter-quantile range) from the hinge. The lower whisker extends from the hinge to the smallest value at most 1.5 * IQR of the hinge. Raw data for the graphs are provided in Source Data File 1. Detailed statistics for each graph are provided in Source Data File 2.

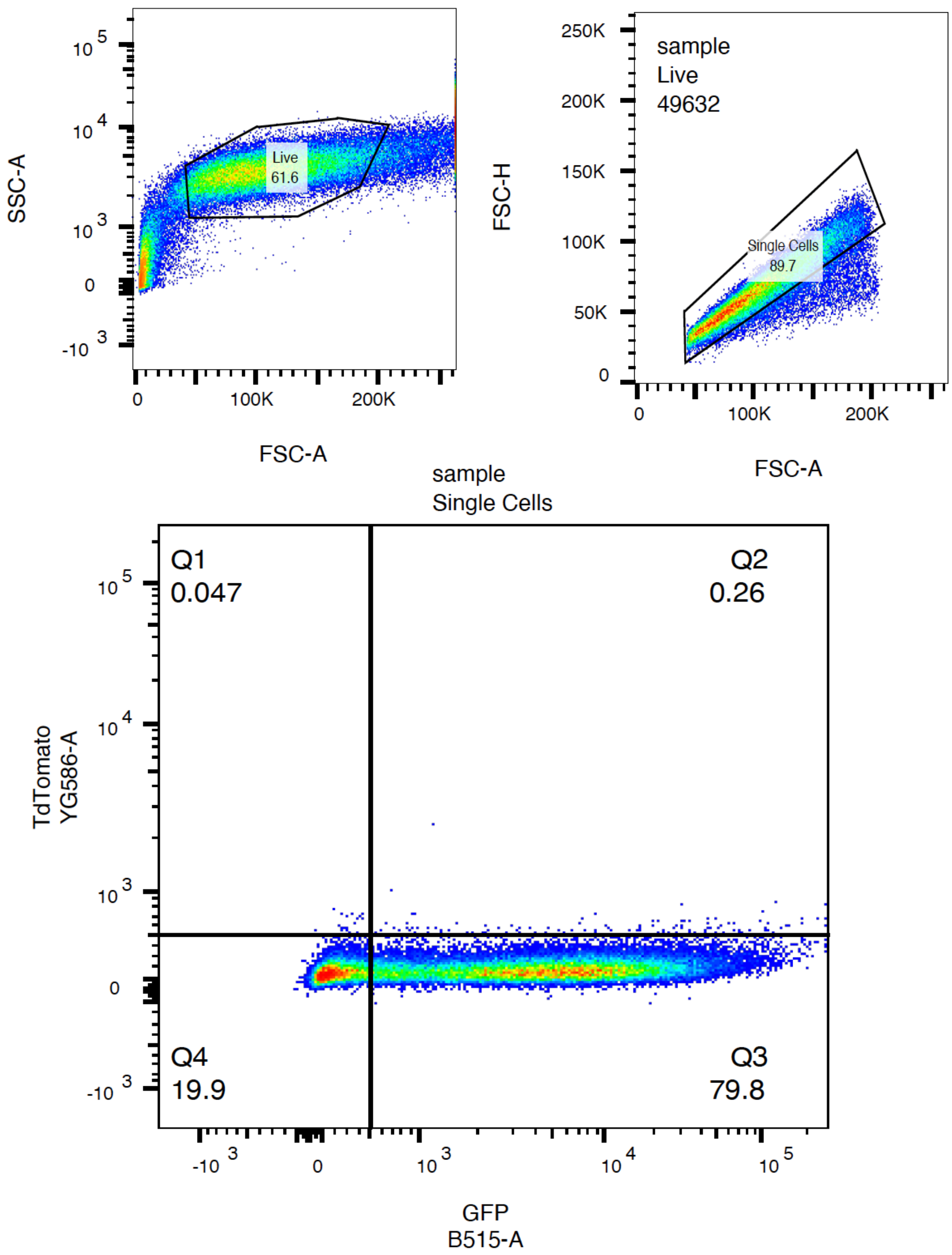
SSC-A
FSC-A
Live
61.6
FSC-H
sample
Live
49632
Single Cells
89.7
FSC-A
sample
Single Cells
TdTomato
YG586-A
Q1
0.047
Q2
0.26
Q4
19.9
Q3
79.8
GFP
B515-A

Supplementary Fig. 9: Example for the FACS gating stragegy. Gating of replica 1 of Huh-7 transduced with LK03-GFP. Gating for live cells, single cells, and GFP expressing cells in Supplementary Figure 1c.
