# Supplementary material for: The AAV capsid can influence the epigenetic marking of rAAV delivered episomal genomes in a species dependent manner
Source: Nat Commun. 2023 Apr 28;14:2448. doi: 10.1038/s41467-023-38106-3 (PMC10147666; doi:10.1038/s41467-023-38106-3)
Supplement: Supplementary file 3 — Description of Additional Supplementary Files Document [file 41467_2023_38106_MOESM3_ESM.pdf]

<u>Description of Additional Supplementary Files</u>

File Name: Supplementary Data 1

Description: Sequences of primers used in qPCR (.xlsx)

File Name: Supplementary Data 2

Description: Vendor source and dilutions of antibodies used in Cut&Tag (.xlsx)

File Name: Supplementary Data 3

Description: Cut&Tag NGS primers (.xlsx)
