# Supplementary material for: The AAV capsid can influence the epigenetic marking of rAAV delivered episomal genomes in a species dependent manner
Source: Nat Commun. 2023 Apr 28;14:2448. doi: 10.1038/s41467-023-38106-3 (PMC10147666; doi:10.1038/s41467-023-38106-3)
Supplement: Supplementary file 7 — Reporting Summary [file 41467_2023_38106_MOESM7_ESM.pdf]

nature portfolio

Corresponding author(s): Mark A. Kay

Last updated by author(s): 2023/03/15

Reporting Summary

Nature Portfolio wishes to improve the reproducibility of the work that we publish. This form provides structure for consistency and transparency in reporting. For further information on Nature Portfolio policies, see our Editorial Policies and the Editorial Policy Checklist.

Statistics

For all statistical analyses, confirm that the following items are present in the figure legend, table legend, main text, or Methods section.

n/a	Confirmed	
☐	☒	The exact sample size (*n*) for each experimental group/condition, given as a discrete number and unit of measurement
☐	☒	A statement on whether measurements were taken from distinct samples or whether the same sample was measured repeatedly
☐	☒	The statistical test(s) used AND whether they are one- or two-sided<br>*Only common tests should be described solely by name; describe more complex techniques in the Methods section.*
☒	☐	A description of all covariates tested
☒	☐	A description of any assumptions or corrections, such as tests of normality and adjustment for multiple comparisons
☐	☒	A full description of the statistical parameters including central tendency (e.g. means) or other basic estimates (e.g. regression coefficient) AND variation (e.g. standard deviation) or associated estimates of uncertainty (e.g. confidence intervals)
☐	☒	For null hypothesis testing, the test statistic (e.g. *F*, *t*, *r*) with confidence intervals, effect sizes, degrees of freedom and *P* value noted<br>*Give P values as exact values whenever suitable.*
☒	☐	For Bayesian analysis, information on the choice of priors and Markov chain Monte Carlo settings
☒	☐	For hierarchical and complex designs, identification of the appropriate level for tests and full reporting of outcomes
☒	☐	Estimates of effect sizes (e.g. Cohen's *d*, Pearson's *r*), indicating how they were calculated

Our web collection on statistics for biologists contains articles on many of the points above.

Software and code

Policy information about availability of computer code

Data collection	Data collection was performed using Bio-Rad ChemiDoc Imaging Systems (vS.2.1 build 11), Tecan i-control™ Microplate Reader Software (v3.4.2), BioRad CFX Maestro (vl.1), BD LSRII Flow Cytometer with BD FACSDiva software v6.1.3, lllumina Hi-Seq 4000, Mi-seq and Nova-seq 6000, Clustal Omega web-based, Swiss-Model, Ami Imaging System
Data analysis	Data analysis was performed using Microsoft Excel (v2111), Quantity One 1-D Analysis (v4.6.8), FlowJo (v10.6.2), GraphPad PRISM software (v9.3.1), SCG cluster, Trimmomatic-0.39, Picard version 2.23, Bowtie2 version 2.2.5, deeptools 3.3, R v4.1, R Gviz 4.1, R ggplot2 4.1.2

For manuscripts utilizing custom algorithms or software that are central to the research but not yet described in published literature, software must be made available to editors and reviewers. We strongly encourage code deposition in a community repository (e.g. GitHub). See the Nature Portfolio guidelines for submitting code & software for further information.

Data

Policy information about availability of data

All manuscripts must include a data availability statement. This statement should provide the following information, where applicable:

- Accession codes, unique identifiers, or web links for publicly available datasets
- A description of any restrictions on data availability
- For clinical datasets or third party data, please ensure that the statement adheres to our policy

All data needed to evaluate the conclusions in the study are present in the main text or the supplementary materials. Request for reagents should be directed to M.A. Kay. All raw and processed sequence data have been deposited in GEO (Gene Expression Omnibus) and are available under accession number GSE226268.

Human research participants

Policy information about studies involving human research participants and Sex and Gender in Research.

Reporting on sex and gender	*Use the terms sex (biological attribute) and gender (shaped by social and cultural circumstances) carefully in order to avoid confusing both terms. Indicate if findings apply to only one sex or gender; describe whether sex and gender were considered in study design whether sex and/or gender was determined based on self-reporting or assigned and methods used. Provide in the source data disaggregated sex and gender data where this information has been collected, and consent has been obtained for sharing of individual-level data; provide overall numbers in this Reporting Summary. Please state if this information has not been collected. Report sex- and gender-based analyses where performed, justify reasons for lack of sex- and gender-based analysis.*
Population characteristics	*Describe the covariate-relevant population characteristics of the human research participants (e.g. age, genotypic information, past and current diagnosis and treatment categories). If you filled out the behavioural & social sciences study design questions and have nothing to add here, write "See above."*
Recruitment	*Describe how participants were recruited. Outline any potential self-selection bias or other biases that may be present and how these are likely to impact results.*
Ethics oversight	*Identify the organization(s) that approved the study protocol.*

Note that full information on the approval of the study protocol must also be provided in the manuscript.

Field-specific reporting

Please select the one below that is the best fit for your research. If you are not sure, read the appropriate sections before making your selection.

☒ Life sciences ☐ Behavioural & social sciences ☐ Ecological, evolutionary & environmental sciences

For a reference copy of the document with all sections, see nature.com/documents/nr-reporting-summary-flat.pdf

Life sciences study design

All studies must disclose on these points even when the disclosure is negative.

Sample size	Sample size was determined by the necessary number of samples needed for sufficient power for statistical testing.<br>For in vitro experiments, majority of the experiments are at least n=3 biological replicas, except Fig 1 e and Extended Data Fig 1 c.<br>For in vivo experiments, n=3 mice or more, size considerations were based upon ability to handle samples for processing and analysis. Sample sizes were determined based up the number needed to test for normalcy, distribution, and significance in order to reasonably determine the likelihood that differences in groups were by chance or by effect. Samples sizes were considered sufficient if the standard deviation within a group was reasonable and the differences between groups great enough to know the effect was unlikely to be a result of random chance.
Data exclusions	No data exclusions were included
Replication	In vitro experiments were performed with at least two technical replicas and three biological replicas. except Fig 1 e and Extended Data Fig 1 c. All attempts at replication were successful for in vitro experiments. Replication of animal experimental findings were primarily determined by five mice, and for AAV-LK03 injections, two independent experiments with orthogonal methods of measurement. All attempts at replication were successful for in vivo experiments.
Randomization	Allocation of samples into groups were random for all non-animal studies. Randomization was based upon animal weight at the beginning of in vivo studies, to ensure average starting weights were equal between groups.
Blinding	Blinding was not performed. Several experiments have been performed and reproduced by S.T and A.G-S.

Reporting for specific materials, systems and methods

We require information from authors about some types of materials, experimental systems and methods used in many studies. Here, indicate whether each material, system or method listed is relevant to your study. If you are not sure if a list item applies to your research, read the appropriate section before selecting a response.

Materials & experimental systems

n/a	Involved in the study	
☐	☒	Antibodies
☐	☒	Eukaryotic cell lines
☒	☐	Palaeontology and archaeology
☐	☒	Animals and other organisms
☒	☐	Clinical data
☒	☐	Dual use research of concern

Methods

n/a	Involved in the study	
☒	☐	ChIP-seq
☐	☒	Flow cytometry
☒	☐	MRI-based neuroimaging

Antibodies

Antibodies used	Cut&Tag<br>anti-Histone H3K4me3 Antibody, SNAP-ChIP Certified (13-0028) (EpiCypher), 0.5ug<br>anti-H3K9me3 polyclonal antibody (C15410193) (Diagenode), 0.5ug<br>anti-Acetyl-Histone H3 (Lys27) Monoclonal Antibody (MA5-23516) (Thermo Fisher), 0.5ug<br>anti-H3K27me3 Monoclonal Antibody Clone G.299.10 (MA5-11198) (Thermo Fisher), 0.5ug<br>anti-Histone H2A antibody - ChIP Grade (ab18255) (Abcam), 0.5ug<br>anti-Histone H3 Antibody ChIP Formulated (26505) (Cell Signaling), 0.5ug<br>anti-Histone H4 antibody - Ch IP Grade (ab10158) (Abcam), 0.5ug<br>Rabbit Anti-Mouse lgG (ab46540) (Abcam), 0.5ug<br>Guinea Pig anti-Rabbit lgG (ABIN101961) (Antibodies Online), 0.5ug
Validation	All antibodies used in the experiment have validation information available on manufacturer's website pages, along with references to peer-reviewed publications.

Eukaryotic cell lines

Policy information about cell lines and Sex and Gender in Research

Cell line source(s)	Huh7 (JCRB0403) cells were purchased from JCRB, 293T (CRL-3216) and Hepa1-6 (CRL-1830), SNU-499 (CRL-2234), PLC (CRL-8024), C3A (CRL-10741), Hepa-1c1c7 (CRL-2026), BNL (CRL-3308), AML12 (CRL-2254) cells were purchased from ATCC.
Authentication	Authentication of cell lines was determined by qPCR analysis of PTGER2 mRNA expression in Huh7 and Hepal-6 cells. 293T cells were authenticated by the ability to produce rAAV vectors.
Mycoplasma contamination	Cell lines were not tested for mycoplasm, as no signs of contamination were ever noted.
Commonly misidentified lines (See ICLAC register)	No commonly misidentified cell lines were used in this study.

Animals and other research organisms

Policy information about studies involving animals; ARRIVE guidelines recommended for reporting animal research, and Sex and Gender in Research

Laboratory animals	Mus musculus, 6 weeks old, male, BALB/c scid mice (Strain #:001803 - The Jackson Laboratory)
Wild animals	No wild animals were used in this study.
Reporting on sex	Mice used were only males
Field-collected samples	No field collected samples were used in this study
Ethics oversight	All animal works were performed in accordance to the guidelines for animal care at Stanford University

Note that full information on the approval of the study protocol must also be provided in the manuscript.

Flow Cytometry

Plots

Confirm that:

☒ The axis labels state the marker and fluorochrome used (e.g. CD4-FITC).

☒ The axis scales are clearly visible. Include numbers along axes only for bottom left plot of group (a 'group' is an analysis of identical markers).

☒ All plots are contour plots with outliers or pseudocolor plots.

☒ A numerical value for number of cells or percentage (with statistics) is provided.

Methodology

Sample preparation	Huh7 or Hepal-6 cultured cells transduced with rAAVs were collected by trypsinization, washed with PBS and resuspended in cold PBS. Cells were kept on ice and protected from light until analyzed.
Instrument	BD LSRII flow cytometer
Software	Data collection was performed using BD FACSDiva software v6.1.3.<br>Data analysis was performed using the FlowJo software (v10.6.2).
Cell population abundance	Collection of data for positive cells of n=10'000
Gating strategy	Singlet cells were gated using FSC/SSC plot and GFP or TdTomato positive populations were determined based on the negative samples which was non-transduced GFP or TdTomato negative cells (Huh7 or Hepal-6)

☒ Tick this box to confirm that a figure exemplifying the gating strategy is provided in the Supplementary Information.
